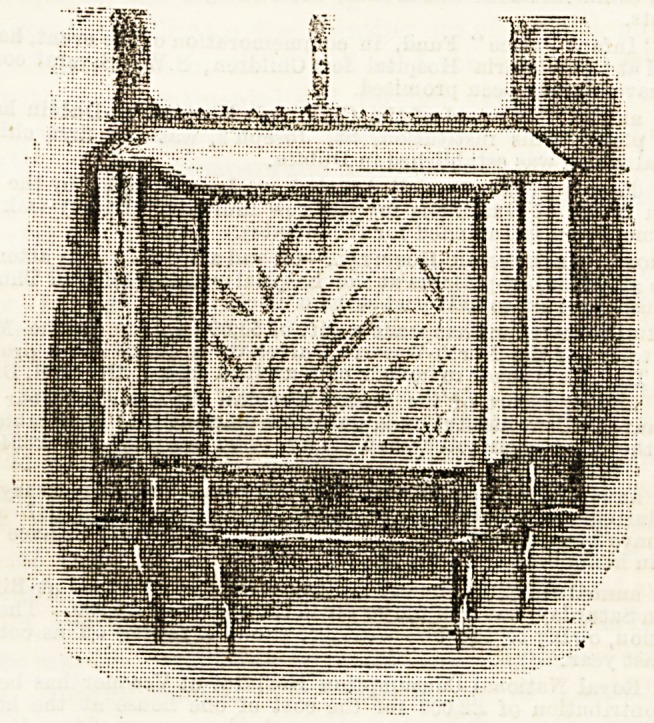# Practical Departments

**Published:** 1894-07-14

**Authors:** 


### PRACTICAL DEPARTMENTS.

### WINDOW GARDENS.

The great importance of providing sick people with surroundings as bright and cheerful as circumstances will permit is being more realised every day, and the pleasant aspect of the wards in most hospitals now-a-days bear eloquent testimony to this fact. Much loving care and thought is expended on the interior decoration of our hospitals, and many are the flowers and pictures which find their way through various channels into the wards, where we may be sure they are of no small use in gladdening and cheering the inmates.

Yet, though so much has been and is being done in this direction, there is plenty of room for further efforts. The particular method of decoration we are thinking of at the moment is—window gardens. Anyone who has seen and noted the charming effect produced by this very simple plan in the children's wards of the London Hospital, must have wondered that the example is not more often followed in cases where ward windows look out upon nothing but an unlovely street or a dead wall, so frequently the only view obtainable in institutions in London and large towns.

Our illustration is of one of these small "gardens" fitted inside the window. Where funds will bear the extra expense it is no doubt a better plan to have the case thrown out from the lower half of the window, thus giving more room for plants and ferns. But this is a more elaborate affair, and the first way answers very well as a rule, at less cost. The frames could be made to fit any window by a local carpenter for a comparatively small sum, which will be well worth the effect obtained.

It is not always easy to keep plants under such conditions in a flourishing and healthy state, but a little care and trouble in attending to them will do wonders in spite of hospital air, and experience soon shows the best kinds to have and the best way to treat them. The boxes from which our illustration is taken are made with the sides to open, and it has been found a good way to leave both sides open for a certain number of hours—all night is a good plan—so as to give plenty of air. Aspidistras are excellent plants for this purpose, and their glossy green and variegated leaves make a substantial background for the "garden."

The refreshing effect of a bit of greenery instead of deadly dull ground glass or an uninviting outside prospect is untold, and besides being a source of real enjoyment to the patients, will prove no less a pleasure to the nurse who makes its preservation her especial business.

### A NEW EXPANDING PORTFOLIO.

We have received from Mr. Honeyman, 37, Great Queen Street, W.C., a portfolio invented by himself, which possesses the great merit of adapting its dimensions to its contents as occasion requires by means of an expanding back. The covers are of board, one being provided with a sort of pocket into which the flexible back piece slips, pulling out to the extent needed as papers increase. The securing piece of elastic or tape is firmly fixed to one cover. It is a very neat, workmanlike case, and will certainly be very useful to many people who have a difficulty in stowing away accumulations of letters and papers. We would only make the suggestion that perhaps it might increase the usefulness of the portfolioif the expanding back could be fastened in some way to prevent it slipping too far out with pressure from inside.

## Figures and Tables

**Figure f1:**